# Polycythaemia Secondary to Hormone Replacement Therapy with Tibolone

**DOI:** 10.1155/2017/3476349

**Published:** 2017-09-27

**Authors:** Laura Staples, Tamara Milder, Philip Young-Ill Choi

**Affiliations:** ^1^Haematology Department, The Canberra Hospital, Canberra, ACT, Australia; ^2^Endocrinology Department, The Canberra Hospital, Canberra, ACT, Australia

## Abstract

We present the case report of a patient with severe polycythaemia associated with tibolone. In our 65-year-old postmenopausal patient who initially presented with haemoglobin 203 g/L [115–160] and haematocrit 0.63 [0.32–0.47], the cessation of tibolone, a synthetic hormone replacement therapy, led to a dramatic and sustained resolution of this patient's polycythaemia to normal haematological values. Tibolone possesses oestrogenic, androgenic, and progestogenic properties. Tibolone therapy may be an infrequently recognized contributor towards polycythaemia in postmenopausal patients presenting to haematology clinics.

## 1. Introduction

Polycythaemia secondary to testosterone replacement therapy has been well described [1]; however we present a case of tibolone-associated polycythaemia.

Tibolone is a synthetic steroid prodrug used to relieve menopausal symptoms and to reduce bone demineralization [2, 3]. It is variably metabolized into three predominant metabolites: *δ*4-isomer and 3*α*- and 3*β*-hydroxytibolone [4]. The *δ*4-isomer has strongest progestogenic and androgenic activity, while 3*β*-hydroxytibolone possesses the most potent oestrogenic activity via oestrogen receptor *α* (ER-*α*) [4].

Tibolone has previously been demonstrated to be associated with minor elevations in Hb and Hct [5]. However, cases of severe polycythaemia have not been reported in the literature to our knowledge.

## 2. Case Presentation

A 65-year-old female was referred to haematology clinic for progressive polycythaemia. She had a past history of hypertension, chronic kidney disease, organic bipolar disorder, and irritable bowel syndrome. She was a chronic smoker of 10 cigarettes per day, on a 30-pack-year history. She denied weight loss, anorexia, dyspnoea, symptoms of obstructive sleep apnoea, symptoms of hyperviscosity, and erythromelalgia.

She had been on tibolone intermittently for over 12 years to treat perimenopausal symptoms. However, she had been regularly taking tibolone 2.5 mg daily for at least 12 months prior to this presentation. Her other medications included nifedipine, reboxetine, and escitalopram, but her adherence to these medications was variable.

On physical examination, the patient appeared mildly flushed but without a ruddy complexion. Her blood pressure was raised to 157/104. Her respiratory examination was normal, and her oxygen saturation was 97% in room air. She weighed 60 kg, and her body mass index was 23. There was no mucosal bleeding. She had no palpable splenomegaly or hepatomegaly.

Her initial investigations were as follows, with reference ranges: haemoglobin (Hb) 203 g/L [115–160], haematocrit (Hct) 0.63 [0.32–0.47], white cell count 7.8 × 10^9^/L [4.0–11.0], and platelet count 200 × 10^9^/L [150–400]. Her blood film demonstrated mild-moderate anisocytosis and occasional target cells but no abnormal white cell or platelet morphology. She had microscopic haematuria on urinalysis and an elevated serum creatinine of 105 *μ*mol/L [45–90].

She was asked to suspend her tibolone and commence low dose aspirin and venesected 450 mL twice while awaiting further investigations. After these venesections, her Hb improved to 182 g/L and Hct 0.54. She continued to smoke regularly.

**Figure 1:**
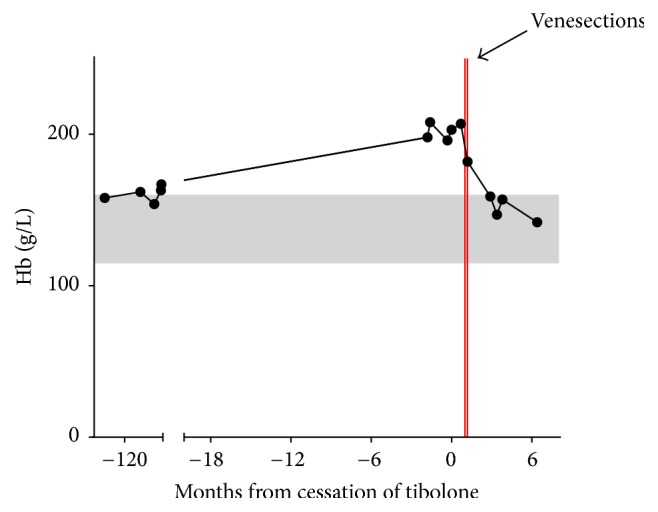
Hb results are depicted from over ten years prior to the cessation of tibolone and for over 6 months after the cessation of tibolone. The grey shaded area represents the reference range for normal values.

Computed tomography and ultrasound imaging of her abdomen excluded renal and hepatic masses but did identify renal calculus disease. JAK2 V617F was negative, and her serum erythropoietin level was 23 U/L [3–24]. No further venesections were performed.  Figure 1 depicts her Hb over time since initial presentation.

Now over six months after ceasing her tibolone, her Hb has fallen to 142 g/L, along with Hct 0.43. There was no evidence of iron deficiency with serum ferritin levels 46 *μ*g/L [20–370] before and 113 *μ*g/L four months after venesection. Faecal occult blood testing has been negative, and she continues to smoke 10 cigarettes per day.

## 3. Discussion

Although cigarette smoking and lung disease are common causes of secondary polycythaemia, there was no change in this patient's pattern of smoking, and there was no evidence of hypoxia. Furthermore, although other medications may be implicated in polycythaemia, this patient was on reboxetine and escitalopram only intermittently, and their previous cessation was not associated with improvement in her Hb or Hct. Likewise, the improvement in polycythaemia after tibolone cessation was not apparently diminished by ongoing nifedipine use.

Other genetic mutations associated with myeloproliferative neoplasia such as Jak2 exon 12, CALR, and MPL mutations were not examined in this case, although the need became less apparent as her polycythaemia improved after tibolone cessation.

In addition to the proandrogenic effects of the *δ*4-isomer metabolite of tibolone, the 3*β*-hydroxytibolone mediated activation of haemopoietic stem cells bearing ER-*α* may participate in stimulation towards enhanced erythropoiesis [4, 6]. Interruption of ER-*α* mediated signaling is associated with impaired erythropoiesis in murine models in female mice but not male mice [6], suggesting a physiological role that may be exaggerated by the administration of exogenous ER-*α* agonists. This may be the mechanism for the observed elevation of Hb and Hct reported in placebo controlled studies with tibolone [5].

Often there may be numerous risk factors that precipitate polycythaemia in susceptible patients, and it is reasonable to exclude them sequentially in order to establish causation. Although the health benefits of smoking cessation are undisputed, in this case it was not necessary to correct the polycythaemia. Thus, this particular case highlights the novel importance of considering hormone replacement therapies as possible contributors to the severity of polycythaemia.

## 4. Conclusion

Polycythaemia is a recognized precipitant for the development of ischaemic vascular events, and we suggest that further studies correlating red cell parameters with patients receiving oestrogen supplements are warranted. This case also highlights the importance of reviewing the use of hormone replacement therapy in older postmenopausal women.
